# Global trends in added sugars and non-nutritive sweetener use in the packaged food supply: drivers and implications for public health

**DOI:** 10.1017/S1368980022001598

**Published:** 2023-05

**Authors:** Cherie Russell, Phillip Baker, Carley Grimes, Rebecca Lindberg, Mark A Lawrence

**Affiliations:** 1 School of Exercise and Nutrition Sciences, Deakin University, 221 Burwood Highway, Geelong, Australia; 2 Institute for Physical Activity and Nutrition, Deakin University, Geelong, Australia

**Keywords:** Sugar, Non-nutritive sweeteners, Nutrition policy, Food policy

## Abstract

**Objective:**

The health implications of excessive added sugar intakes have led to national policy actions to limit their consumption. Subsequently, non-nutritive sweeteners (NNS) may be used to maintain product sweetness. We aimed to assess trends in quantities of added sugars and NNS sold in packaged food and beverages worldwide, and the association between these trends and the number of national policy actions across regions to reduce added sugar consumption.

**Design:**

(i) Longitudinal analysis of Euromonitor sales data (2007–2019) to assess the quantity of added sugars (kg) and NNS (g) sold in packaged foods and beverages globally, across regions, and across country income categories; (ii) policy-mapping of policy actions targeting added sugar consumption globally from the NOURISHING database; and (iii) Spearman’s correlations to assess the association between national policy actions across regions and changes in added sugar/NNS sales.

**Setting:**

Worldwide.

**Participants:**

Not applicable.

**Results:**

Per capita volumes of NNS from beverage sales increased globally (36 %). Added sugars from beverage sales decreased in high-income countries (22 %) but increased in upper-middle-income countries (UMIC) and lower-middle-income countries (LMIC) (13–40 %). Added sugars from packaged food sales increased globally (9 %). Regions with more policy actions had a significant increase in NNS quantities from beverage sales (*r* = 0·68, *P* = 0·04). The sweetness of the packaged food supply (the sweetness of each NNS and added sugar, relative to sucrose, multiplied by sales volume) increased over time.

**Conclusions:**

The increasing use of NNS to sweeten beverages globally, and in packaged food in UMIC and LMIC, may have health and dietary implications in the future. Their use as a substitute for added sugar should be considered in public health nutrition policymaking.

Human beings have evolved with a biological preference for sweetness^(1)^. Sweet foods were likely an important source of energy when food was scarce^(2)^. Subsequently, the food industry has increasingly saturated the market in high-income countries (HIC) with palatable and potentially addictive products sweetened with added sugars^(3–5)^. Added sugars are defined as mono- and disaccharides added to foods during processing and preparation^(6)^. This trend is also increasingly evident in middle-income countries and low-income countries, as part of the ‘nutrition transition’^(3,4)^. Transnational food and beverage corporations (i.e. enterprises involved in the global production and distribution of foods and beverages) contribute to this transition through their marketing of unhealthy foods in pursuit of growth opportunities in emerging markets experiencing rapid economic growth, market liberalization and urbanization^(3,4)^. The increasing sweetness of diets globally may play a critical role in shaping taste preferences, food choices, and consequent health outcomes^(5,7)^.

Overconsumption of added sugars is a recognized dietary risk factor for the global burden of disease and is associated with obesity, type 2 diabetes, and dental caries, particularly when consumed from sugar-sweetened beverages (SSB)^(8,9)^. The WHO recommends that free sugar consumption (consisting of both added and naturally occurring sugars in honey, syrups and fruit juices) is limited to 10 % of total energy intake^(10)^. Despite this, added sugar consumption alone contributes on average 11–17 % and 7–11 % of energy intake in European children and adults, respectively^(11)^, and 8–20 % across the US population^(12)^. This overconsumption of added sugars has led to international policy actions to limit their use by food manufacturers and consumers, including SSB taxes, education campaigns, advertising restrictions and front-of-pack labeling^(13)^.

Several of these policies encourage the reformulation of packaged foods to lower their added sugar composition^(13)^. Reformulation may involve a reduction in added sugars without replacement, making products less sweet. Often, however, added sugars are partially or completely substituted with non-nutritive sweeteners (NNS), defined as substances with little to no energy which impart sweetness^(14)^. Despite their lack of calories, NNS have other biological effects, the health impacts of which are contested. Clinical trials have demonstrated a reduction in BMI and fasting blood glucose associated with NNS consumption^(15,16)^. However, observational studies have reported associations between NNS consumption and weight gain^(17,18)^, changes to the gut microbiome^(18,19)^ and type 2 diabetes^(18,20)^.

Concerns have also been raised regarding the long-term impacts of NNS consumption on dietary balance, sustainability, and population health. First, habitual NNS consumption may contribute to shifting population palates, encouraging sweet taste preferences^(18)^. Second, ultra-processed foods (UPF) that contain NNS may receive a ‘health halo’ and displace nutritious whole foods from the diet. UPF are industrial formulations which typically contain cosmetic and other types of additives^(21)^. These products are designed to be hyper-palatable, affordable, and convenient and are often marketed intensively^(21)^. UPF are markers of poor diets^(22)^ and have known adverse health^(23)^ and environmental^(24)^ impacts. Finally, certain NNS are considered environmental contaminants (sucralose and acesulfame K) and are not effectively removed from wastewater^(25)^.

Previous research suggests that NNS are used as ingredients in a variety of products, though most frequently in beverages, dairy products and confectionary^(13)^. However, few studies have examined population-level NNS consumption over time, though increased intakes have been observed in Europe and the USA^(13)^. Additionally, the longitudinal trends of NNS use in specific product categories, and the differences in their use between regions and countries, have not been explored in previous literature. Thus, the aims of this study are to evaluate the longitudinal trends in worldwide quantities of added sugars and NNS sold in packaged food and beverages and assess the association between these trends and the number of national policy actions in each region to reduce added sugar consumption. We address these aims by answering the following questions: first, how has the use of added sugars and NNS in packaged food and beverages varied over time globally, in different geographical regions, and in different country income categories? Second, do these trends differ in regions with implemented national policy actions to reduce added sugar consumption? Third, has there been an overall increase or decrease in the sweetness of the global packaged food and beverage supply?

## Methods

We undertook a quantitative analysis of global, regional and country income category trends of the apparent consumption of added sugar and NNS using per capita market sales data and mapping of added sugar policies.

### Data collection

#### Sugar and non-nutritive sweetener intakes

Globally comparable, nationally representative, longitudinal household food expenditure or individual food intake survey data for added sugars and NNS were unavailable. Instead, we used country-level sales volume data (the quantity in kilograms sold through combined retail and food service channels) from the Euromonitor Passport database^(26)^ for the years available (2007–2019), with projections to 2025. Passport data are collected from trade associations, industry bodies, business press, company financial reports, company filings and official government statistics. Projections are based on Euromonitor Passport macroeconomic modeling, which accounts for economic and social trends^(26)^. Though these data have limitations, including the omission of food sold through informal channels and the constraints of using sales data as a proxy for consumption, this database has been used in similar analyses^(4,27–29)^ and is not biased by participant recall.

The quantity of added sugars and NNS sold in packaged food and beverages were available for eighty countries, which were subsequently classified by World Bank Country and Lending Group Regional classifications and Income Category classifications^(30)^ (see online Supplemental 1). This included thirty-nine HIC, twenty-four upper-middle-income countries (UMIC) and sixteen lower-middle-income countries (LMIC), constituting 61·9 %, 38·1 % and 25·4 % of the sample, respectively. Low-income countries were excluded from the analysis due to a lack of available data. Taiwan was also excluded from the analysis as population data were not available. Product categories included in the analysis, as defined in the Passport database, are shown in Table 1.

**Table 1:** Product categories used in the analysis, as defined in the Euromonitor Passport database

Category	Subcategory
Packaged foods	Sauces, dressings and condiments
	Ready meals
	Soup
	Sweet spreads
	Baby food
	Dairy products
	Confectionary
	Ice cream and frozen desserts
	Savory snacks
	Sweet biscuits, snack bars and fruit bars
	Baked goods
	Breakfast cereals
	Processed fruit and vegetables
	Processed meat and seafood
	Meat substitutes
	Rice, pasta and noodles
Beverages	Bottled water
	Carbonates
	Concentrates
	Juice
	Ready-to-drink coffee
	Ready-to-drink tea
	Energy drinks
	Sports drinks
	Asian speciality drinks*

* Asian specialty drinks – as defined by passport, this category includes all traditional Asian drinks not included in ready-to-drink tea or juice drinks, including Bandung, bird’s nest, tamarind juice, ginger, lemongrass, roselle, zalaka, jelly drinks, sugar cane and vinegar drinks, among others.

### Sweetener classification

For the purposes of this study, sugar alcohols and other novel low-calorie sweeteners were included in the category of NNS due to their negligible contribution to energy intake. We categorized sweeteners based on the Passport database classifications as shown in Table 2, including their relative sweetness to sucrose^(13)^. Despite fruit concentrate being classified as an added sugar in previous studies and some national dietary guidelines, we excluded ‘fruit’ as an added sugar in this study as the Passport data did not differentiate between whole fruit and fruit concentrate. Unfortunately, not all added sugars and NNS used in the global food supply had available data for each country, year or food/beverage category. For example, data on stevia sales volume were not available for some countries (*n* 35), despite being the fastest growing NNS in several regions^(31,32)^. Thus, the quantities reported in our study for each sweetener category may be under-representative of true levels.

**Table 2:** Sweetener categories captured in the analysis

Sweetener category	Ingredient	Relative sweetness to sucrose	
Added sugars	Brown sugar	1·0	
	Dextrose	0·8	
	Fructose	1·7	
	Fruit juice	1·1*	
	Glucose/corn syrup	1·1	
	Glucose/fructose syrup	1·1	
	High fructose corn syrup	1·1	
	Honey	1·0	
	Invert sugar	1·0	
	Isomalt	0·5	
	Isomaltulose	0·4	
	Lactose	0·2	
	Malt	0·5	
	Malt extract	0·5	
	Maltodextrin	0·2	
	Maltose syrup	0·5	
	Molasses	0·9	
	Oligofructose	0·4	
	Polydextrose	0·1	
	Sucrose	1	
	Treacle	0·9	
Non-nutritive sweeteners (including sugar alcohols)	Acesulfame K	200	
	Aspartame	175	150–200†
	Cyclamate	40	30–50
	Erythritol	0·7	
	Maltitol	0·9	
	Maltitol syrup	0·9	
	Mannitol	0·6	
	Saccharin	400	300–500
	Sorbitol	0·6	
	Stevia	300	250–350
	Sucralose	600	550–750
	Xylitol	1·0	

* The relative sweetness of the average of apple and orange juice was used for all fruit juices in the analysis, as the most used juice in manufactured products^(35)^.

† For all non-nutritive sweeteners with a range of sweetness relative to sucrose, both the midpoint and range are shown.

#### Policy context

We used the search term ‘sugar’ to source country-specific, government-implemented policy actions to reduce added sugar intakes from the World Cancer Research Fund (WCRF)’s NOURISHING database for 2007–2019^(33)^. Data were included only for the countries (or states, in the case of the USAs) with available NNS/added sugar data. Only currently implemented policy actions were included. Policy actions that were implemented prior to 2007 but with updated terms (such as an increased rate of taxation) were also included. Country data were then grouped by World Bank Country and Lending Groups Regional classifications (see online Supplemental 1).

### Data analysis

We converted total sales volume data to either kg/capita or g/capita (for added sugar and NNS, respectively) for each region, income category and time period using population data from the World Bank’s World Development Indicators database^(34)^. Given the disparity of quantities needed to sweeten products, added sugar quantities are presented as kg/capita, while NNS quantities are presented as g/capita. Each sweetener under study has a different sweetness threshold; thus, their contribution to the sweetness of the food supply cannot be determined based only on the quantity used. Therefore, to determine how much each sweetener category has contributed to the overall sweetness of the global packaged food and beverage supply, we multiplied the sales volume in kg/capita of each sweetener by their relative sweetness to sucrose, which is the universal baseline for sweetness (Table 2). The relative sweetness of apple and orange juice was used as an average for all fruit juices in the analysis, as these are the most used in manufactured products^(35)^. For NNS with a range of sweetness relative to sucrose, the midpoint was used in the analysis.

Nationally implemented policy actions to reduce added sugar consumption were grouped by World Bank Country and Lending Groups Regional classifications and tabulated. The association between the number of policy actions in each region (in total and across policy category) in 2019 and the change in the amount of added sugars and NNS in each region were determined using a Spearman correlation test. A *P*-value of <0·05 was considered significant. Analytical and descriptive statistics (percentage change, ratio changes and averages) and graphical outputs to represent the data were generated using Microsoft Excel (V.2108).

## Results

Percentage changes in per capita quantities of both added sugars (kg) and NNS (g) sold in beverages and packaged food from 2007 to 2019 for all regions and country income categories (Table 3), and the changes in the ratio of added sugar and NNS quantities in each region and income category (Table 4), are discussed in detail below.

**Table 3:** Percent change in added sugar and non-nutritive sweetener volumes sold in packaged foods and beverages from 2007 to 2019 in regional and country income categories

Region/income category	Sales volume 2007	Sales volume 2019	% Change
Beverages			
Added sugars (kg/capita)			
World	9·4	8·3	↓ 12
East Asia and the Pacific	5·0	5·7	↑ 14
Europe and Central Asia	21·3	16·8	↓ 21
Latin America and the Caribbean	12·3	12·7	↑ 3
Middle East and North Africa	4·9	5·0	↑ 2
North America	58·9	42·7	↓ 28
South Asia	0·7	1·6	↑ 129
Sub-Saharan Africa	1·4	1·8	↑ 39
HIC	35·5	27·7	↓ 22
UMIC	7·1	8·0	↑ 13
LMIC	1·6	2·4	↑ 50
Non-nutritive sweeteners (g/capita)			
World	6·1	8·3	↑ 36
East Asia and the Pacific	1·5	2·2	↑ 47
Europe and Central Asia	14·3	16·8	↑ 17
Latin America and the Caribbean	7·7	8·9	↑ 16
Middle East and North Africa	1·9	1·9	n/a
North America	26·3	57·5	↑ 119
South Asia	0·3	0·7	↑ 133
Sub-Saharan Africa	0·0	0·0	n/a
HIC	19·4	28·8	↑ 48
UMIC	3·4	4·3	↑ 26
LMIC	1·0	1·0	↑ 0
Packaged food			
Added sugars (kg/capita)			
World	5·7	6·2	↑ 9
East Asia and the Pacific	4·4	6·1	↑ 39
Europe and Central Asia	13·6	13·8	↑ 1
Latin America and the Caribbean	7·2	7·3	↑ 1
Middle East and North Africa	2·5	3·1	↑ 24
North America	26·5	25·5	↓ 4
South Asia	0·9	1·6	↑ 78
Sub-Saharan Africa	0·6	0·6	n/a
HIC	18·4	18·2	↓ 1 %
UMIC	5·6	7·3	↑ 30
LMIC	1·4	2·0	↑ 43
Non-nutritive sweeteners (g/capita)			
World	63·2	65·2	↑ 3
East Asia and the Pacific	43·8	54·7	↑ 25
Europe and Central Asia	144·1	154·3	↑ 7
Latin America and the Caribbean	83·1	79·0	↓ 5
Middle East and North Africa	20·6	25·8	↑ 25
North America	379·9	360·6	↓ 5
South Asia	6·5	10·6	↑ 65
Sub-Saharan Africa	0·0	0·0	n/a
HIC	249·4	238·5	↓ 4
UMIC	47·3	61·1	↑ 29
LMIC	16·3	22·8	↑ 40

HIC, high-income countries; UMIC, upper-middle-income countries; LMIC, lower-middle-income countries.

**Table 4:** Ratio of added sugar (kg) to non-nutritive sweetener volumes (g) by country income category from 2007 to 2019 for beverages and packaged foods

Region/income category	Ratio of added sugar (kg) to non-nutritive sweetener (g)		% Change
	2007	2019	
Beverages			
World	1·2	1·0	↓20
East Asia and the Pacific	2·5	2·9	↑40
Europe and Central Asia	1·7	1·0	↓70
Latin America and the Caribbean	1·5	1·4	↓10
Middle East and North Africa	2·5	2·5	n/a
North America	2·3	0·7	↓160
South Asia	0·6	1·6	↑100
Sub-Saharan Africa	n/a	n/a	n/a
HIC	1·9	1·0	↓90
UMIC	2·1	1·9	↓11
LMIC	1·6	2·4	↑33
Packaged food			
World	0·1	0·1	n/a
East Asia and the Pacific	0·0	0·0	n/a
Europe and Central Asia	0·1	0·1	n/a
Latin America and the Caribbean	0·1	0·1	n/a
Middle East and North Africa	0·1	0·1	n/a
North America	0·1	0·1	n/a
South Asia	0·2	0·2	n/a
Sub-Saharan Africa	n/a	n/a	n/a
HIC	0·1	0·1	n/a
UMIC	0·1	0·1	n/a
LMIC	0·1	0·1	n/a

HIC, high-income countries; UMIC, upper-middle-income countries; LMIC, lower-middle-income countries.

### Global trends in the kg/capita of added sugars and g/capita of non-nutritive sweeteners sold in beverages

#### Regional trends

**Fig. 1:**
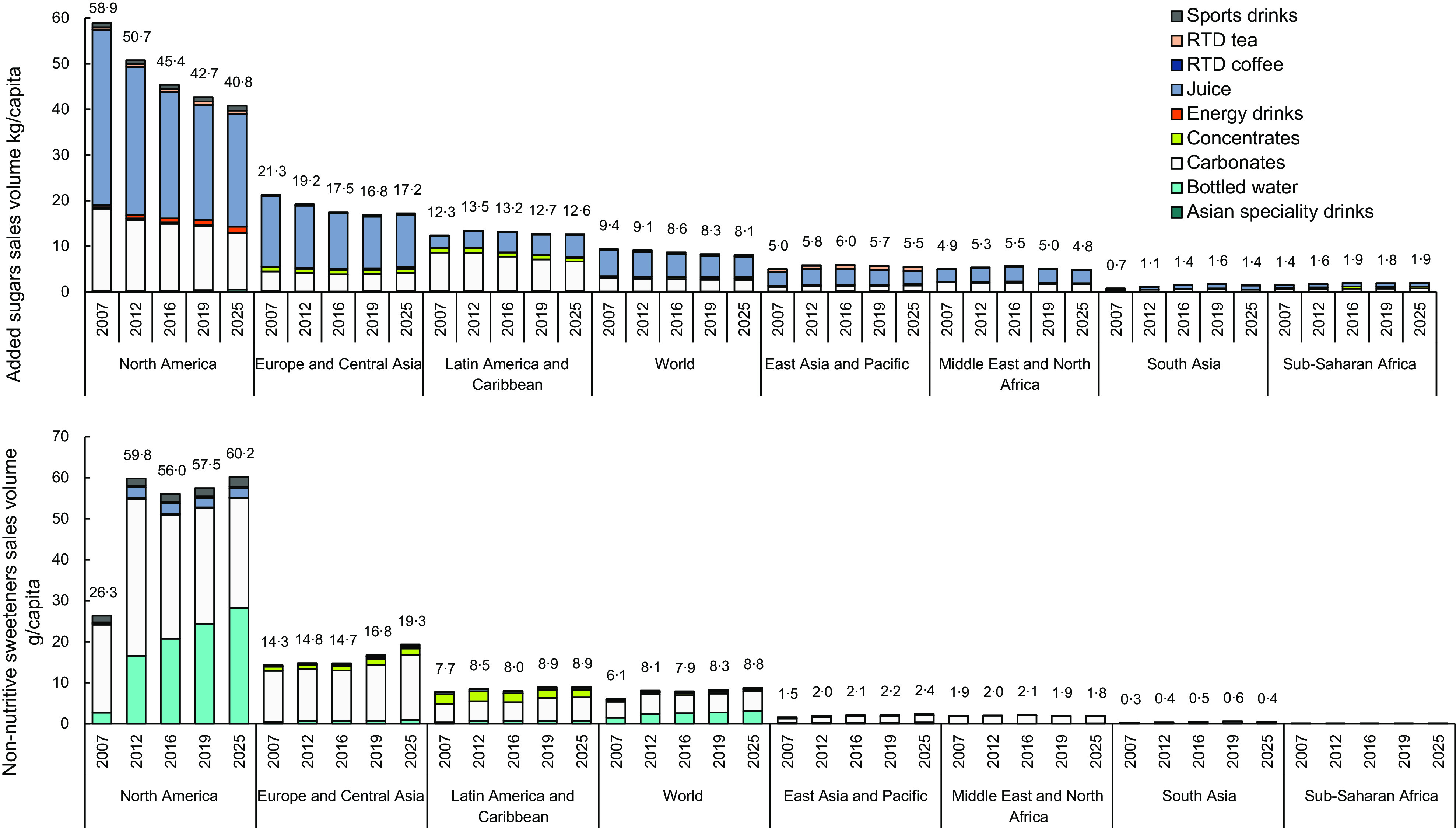
Added sugar (kg/capita; top) and non-nutritive sweeteners volumes (g/capita; bottom) sold in beverages by region, 2007–2019 with projections to 2025

Trends in per capita quantities of added sugars and NNS sold in beverages from 2007 to 2019 for each region are shown in Fig. 1.

The per capita quantity of added sugars sold in beverages has decreased globally from 2007 to 2019 (1·1 kg/capita, 12 %), predominantly in the regions of North America and Europe and Central Asia, with decreases of 16·2 kg/capita (28 %) and 4·5 kg/capita (21 %), respectively. Despite this decrease, the total kg/capita for each year was still markedly higher in North America than other regions, with global changes reflective of trends in this region. Global decreases in added sugar quantities sold in beverages were predominantly from juice, with decreases of 1 kg/capita globally and 13·3 kg/capita in North America. Added sugar quantities supplied by carbonates also decreased by 0·4 kg/capita globally and 3·8 kg/capita in North America. Comparatively, the per capita quantity of added sugars sold in beverages increased in all other regions, though by less than 1 kg/capita (2 %–129 %).

The g/capita of NNS sold in beverages increased in all regions except the Middle East and North Africa and sub-Saharan Africa. Growth between 2007 and 2019 was highest in North America, with an increase of 31·2 g/capita (119 %). Increases in NNS quantities were predominantly from bottled water (21·7 g/capita in North America and 1·3 g/capita globally) and carbonates (6·6 g/capita in North America and 0·7 g/capita globally). The ratio of added sugar to NNS quantities supplied by beverage sales decreased globally in Europe and Central Asia, Latin American and the Caribbean, the Middle East and North Africa, and North America. This suggests that the amount of NNS sold per kg of added sugars increased. Comparatively, the ratio of added sugar to NNS increased in East Asia and the Pacific, and South Asia, suggesting that amount of NNS sold per kg of added sugars decreased.

#### Country income category trends

Trends in the quantity of added sugars and NNS sold in beverages per capita from 2007 to 2019 for each country income category are shown in Supplemental 2. Between 2007 and 2019, the per capita quantity of added sugars sold in beverages has decreased in HIC (7·8 kg/capita, 22 %) though increased in UMIC and LMIC, with growth of 0·9 kg/capita (13 %) and 0·8 kg/capita (50 %), respectively. Comparatively, the quantity of NNS per capita from beverage sales between 2007 and 2009 has increased in all country income categories, with the most growth seen in HIC (9·5 g/capita, 48 %). The ratio of added sugar to NNS quantities sold in beverages decreased from 1·9 to 1·0 kg sugar/g NNS in HIC and from 2·1 to 1·9 kg sugar/g NNS in UMIC, suggesting that amount of NNS sold per kg of added sugars increased. Comparatively, the ratio of added sugar to NNS quantity increased from 1·6 to 2·4 kg sugar/g NNS in LMIC, suggesting that amount of NNS sold per kg of added sugars decreased.

### Global trends in the kg/capita of added sugars and g/capita of non-nutritive sweeteners sold in packaged food

#### Regional trends

**Fig. 2:**
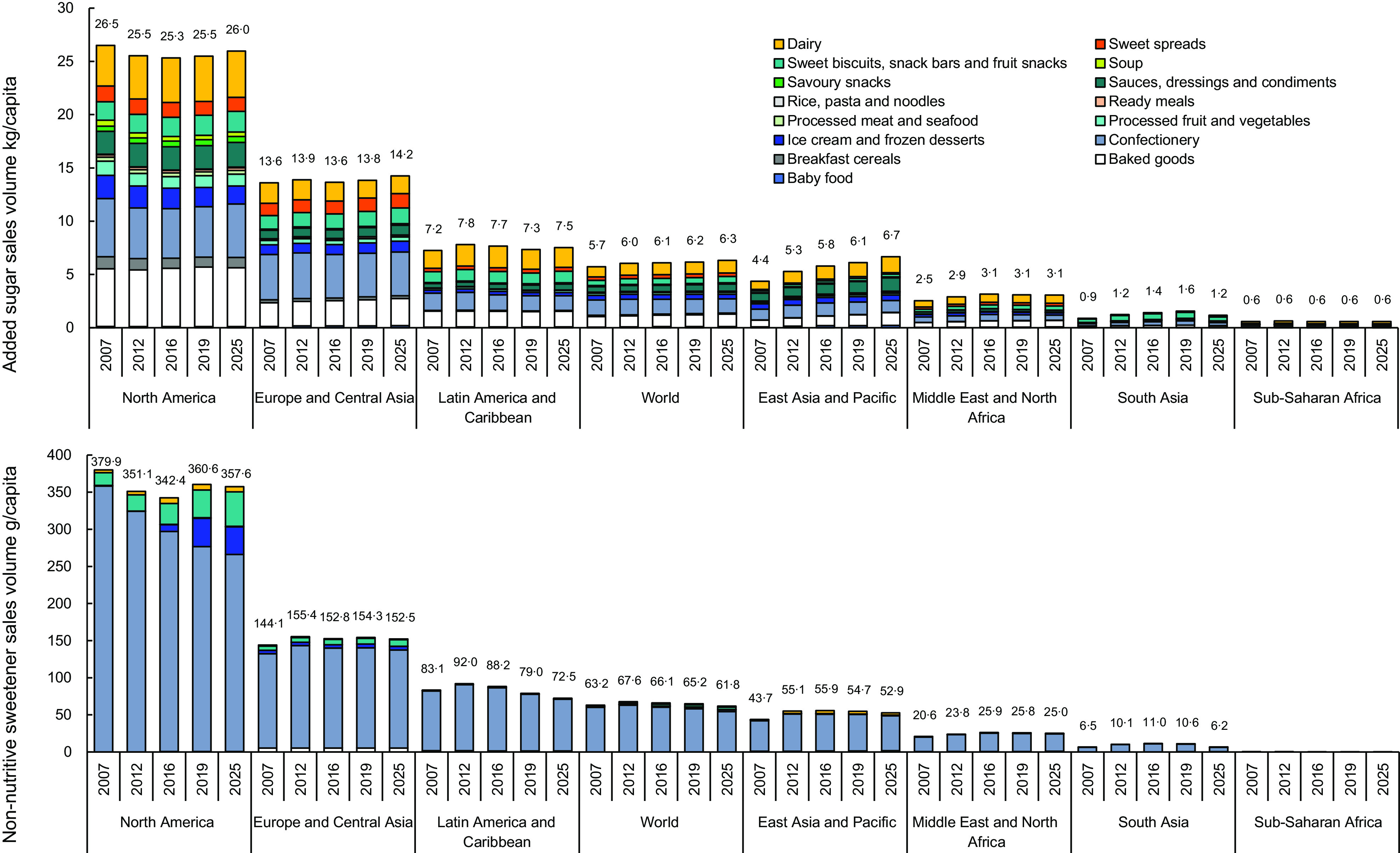
Added sugar (kg/capita top) and non-nutritive sweeteners volumes (g/capita bottom) sold in packaged foods for each region, 2007–2019 with projections to 2025

Trends in the quantity of added sugars and NNS sold in packaged food per capita from 2007 to 2019 for each region are shown in Fig. 2.

Between 2007 and 2019, the per capita trends in the quantity of added sugars and NNS sold in packaged foods increased globally (0·5 kg/capita, 9 %) in East Asia and Pacific, Europe and Central Asia, Latin America and the Caribbean, the Middle East and North Africa, and South Asia. However, increases in these regions were small, ranging from 0·1 to 1·7 kg/capita. A small decrease in quantity was observed in North America (1·01 kg/capita). All increases and decreases in the quantity of added sugars supplied by each packaged food category under study were less than 0·4 kg/capita.

Per capita quantities of NNS sold in packaged foods from 2007 to 2019 increased globally, though by only 2·0 g/capita (3 %). Per capita quantities increased in Europe and Central Asia, East Asia and the Pacific, the Middle East and North Africa, and South Asia, with increases from 4·2 to 13·8 g/capita. Quantities initially increased in Latin America by 10 % from 2007 to 2012, followed by a net decrease of 4·1 g/capita (5 %) by 2019. Similarly, North America had an initial decrease in g/capita from 2007 to 2016, followed by an increase in 2019, though with an overall net decrease of 19·4 g/capita (5 %). Despite being the most prolific source of NNS in the data, the amount of NNS supplied by confectionary sales decreased from 2007 to 2019 by 2·1 g/capita (3 %) globally and 82·3 g/capita (23 %) in North America. The ratio of added sugar to NNS quantities supplied by packaged food remained stable in all regions.

#### Country income category trends

Trends in the per capita quantity of added sugars and NNS sold in packaged food from 2007 to 2019 for each country income category are shown in Supplemental 3. Between 2007 and 2019, the per capita amount of added sugars sold in packaged foods remained relatively stable in HIC (0·2 g/capita, 1 %), though increased in UMIC and LMIC, with growth of 1·7 g/capita (30 %) and 0·6 g/capita (43 %) respectively. Similarly, the quantity of NNS per capita from packaged food sales for the same period decreased for HIC (10·9 g/capita, 4 %), though increased for both UMIC (13·8 g/capita, 29 %) and LMIC (6·5 g/capita, 40 %). The ratio of added sugar to NNS quantities supplied by food remained stable.

### Global trends in sales of various non-nutritive sweetener

**Fig. 3:**
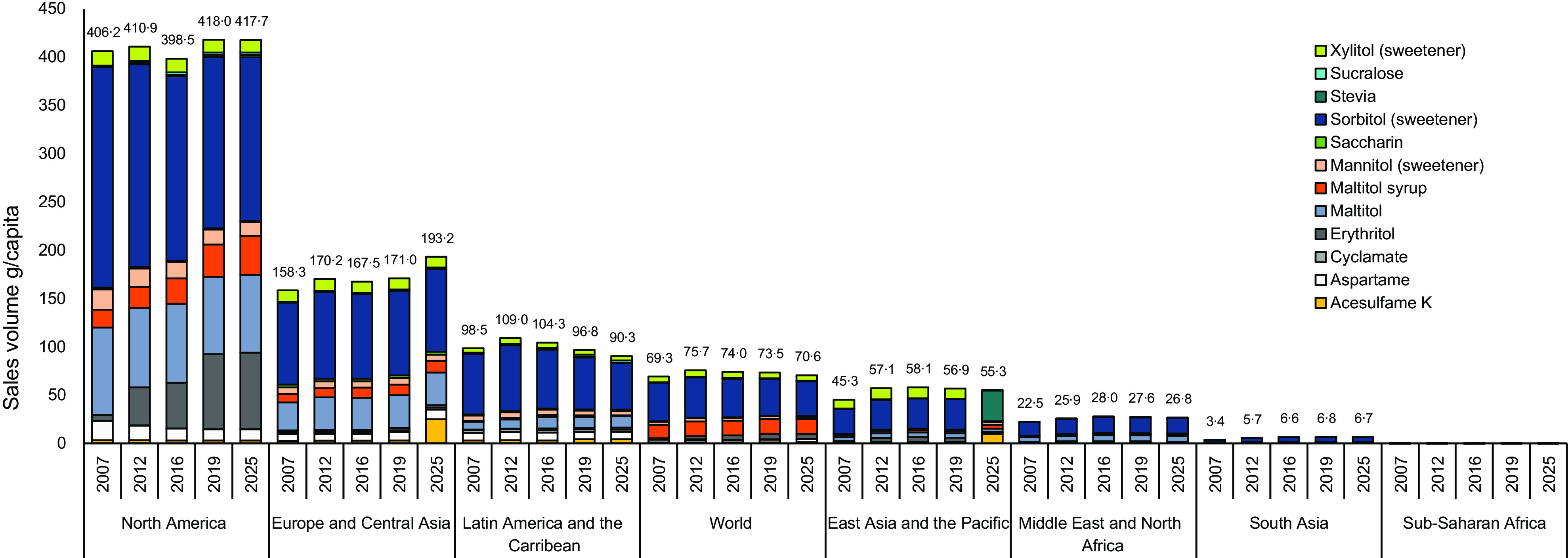
Non-nutritive sweetener volumes (g/capita) sold in packaged foods and beverages for each region, 2007–2019 with projections to 2025

Of the NNS captured in this study, sorbitol had the highest g/capita supplied by packaged foods and beverages from 2007 to 2019 for all regions and income categories, though amounts decreased over time (Fig. 3, see online Supplemental 4). Stevia had the highest rates of growth in all regions, with a global increase of 10 599 %, though the volume of growth was small (0·2 g/capita). Erythritol had the second highest rate of growth in most regions and income categories (ranging from 1145 % in North America to 0 % in sub-Saharan Africa), followed by sucralose (ranging from 189 % in Latin America to 18 % in the Middle East and North Africa). East Asia and the Pacific and South Asia had growth in all NNS sales volumes.

### Changes in total sweetness of beverages and packaged foods

**Fig. 4:**
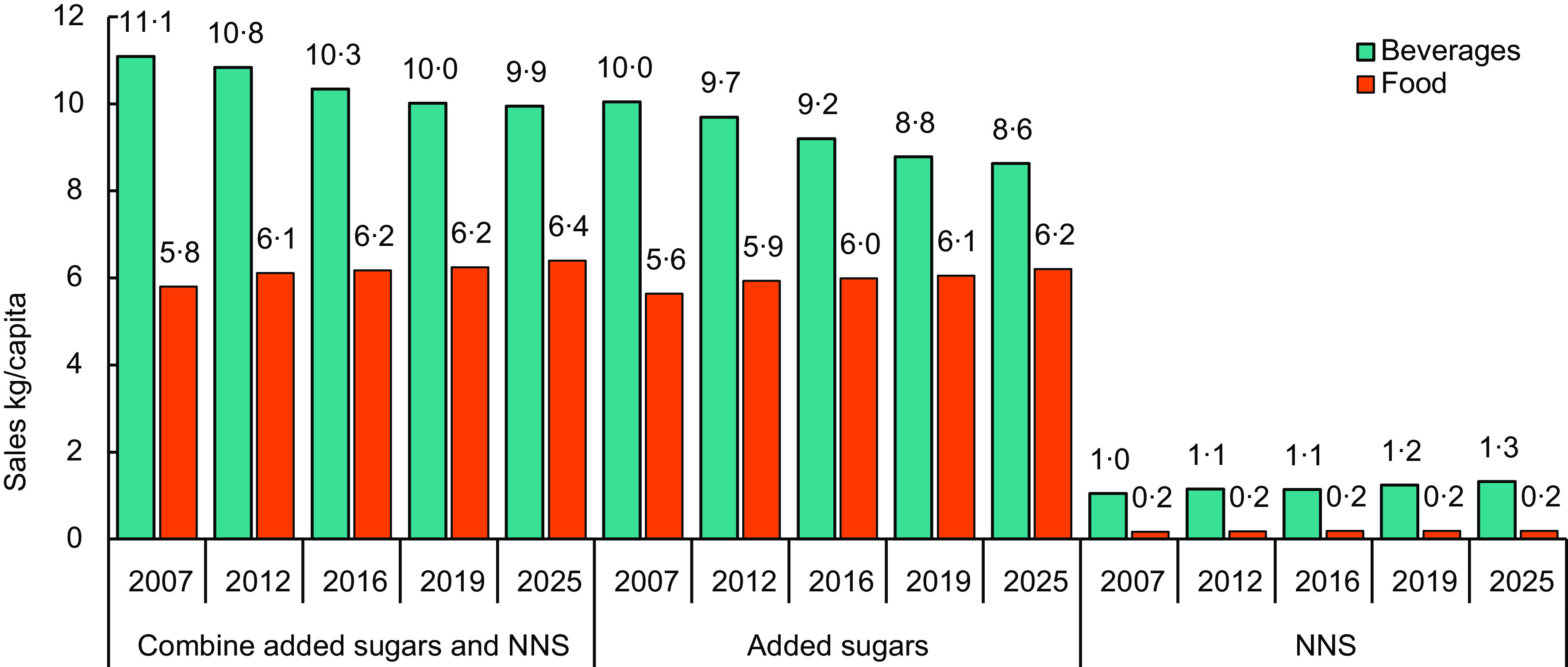
Global volume of sweeteners (kg/capita) sold in packaged food and beverages, adjusted for relative sweetness to sucrose, for 2007–2019 with projections to 2025. NNS, non-nutritive sweetener

After converting the g/capita sales of all sweeteners to their relative sweetness to sucrose, the global quantity of added sugars and NNS combined (or the total ‘sweetness’) from beverage sales decreased (1·1 kg/capita, 9·7 %) in the period 2007–2019. Comparatively, the global quantity of added sugars and NNS combined (or the total ‘sweetness’) from packaged food sales over the same period increased (0·4 kg/capita, 7·6 %) (Fig. 4). This suggests that globally, the level of sweetness of beverages is decreasing over time, while the level of sweetness of packaged foods is increasing. The proportion of sweetness supplied by added sugars sold in beverages decreased (1·3 kg/capita, 12·6 %), while the proportion supplied by NNS increased (0·2 kg/capita, 18·8 %). Comparatively, the sales of sweetness supplied by added sugars in packaged foods increased (0·4 kg/capita, 12·6 %), while the sales supplied by NNS remained stable.

### Global policy actions to reduce added sugar consumption

**Fig. 5:**
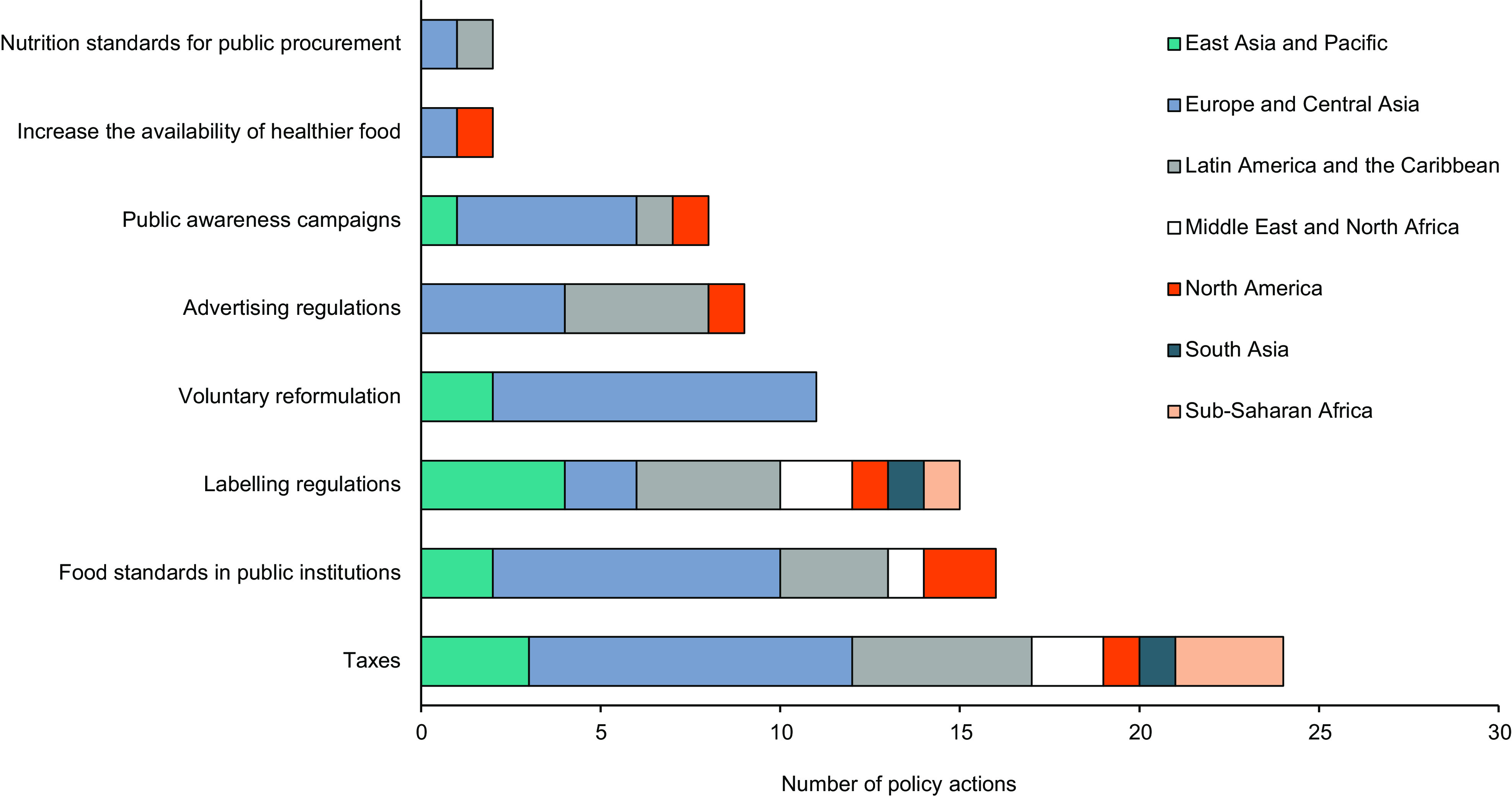
Number of implemented policy actions that may reduce added sugar consumption in each region

A summary of policy actions implemented between 2007 and 2019 globally that may reduce added sugar consumption, either by targeting added sugars specifically or indirectly influencing their use in the food supply, is shown in Fig. 5. The most prolific policy action was taxation of SSB (*n* 22), followed by the implementation of food standards in public institutions, such as hospitals and public schools (*n* 16), and labeling regulations (*n* 15). The regions with the most policy actions included Europe and Central Asia (*n* 40), Latin America and the Caribbean (*n* 18), and East Asia and the Pacific (*n* 12).

Overall, there was no association between the total number, or specific type, of policy actions targeting sugar reduction in each region and the amount of added sugar sold in packaged food or beverages (see online Supplemental 5). One exception was a significant negative correlation between the number of policy actions targeting the increased availability of healthy food and added sugar quantities sold in beverages (*n* 5, *r* = −0·79, *P* = 0·02). Comparatively, the total number of policy actions implemented in each region was significantly correlated with greater NNS quantities sold in beverages (*n* 5, *r* = 0·68, *P* = 0·04), but not packaged food. Furthermore, a higher number of specific policy actions for ‘advertising regulations’ (*n* 5, *r* = 0·78, *P* = 0·02), food standards in public institutions’ (*n* 5, *r* = 0·73, *P* = 0·03) and an ‘increased availability of healthy food’ (*n* 5, *r* = 0·79, *P* = 0·02) was significantly correlated with a greater quantity of NNS sold in beverages.

## Discussion

The aim of this study was to assess the longitudinal trends in worldwide quantities of added sugars and NNS supplied by packaged food and beverage sales, and the association between these trends and regional policy actions. Here, we critically discuss the changes in added sugar and NNS amounts sold in the packaged food and beverage supply, the overall sweetness of the food and beverage supply, policy actions to reduce added sugar consumption, and other potential drivers of NNS use.

### Food supply transitions – how has sweetener use changed over time?

Per capita quantities of NNS sold in beverages are increasing globally, particularly in carbonates. This likely reflects that most implemented policy actions to reduce added sugar consumption have been SSB taxes, which in part aim to incentivise manufacturers to reformulate their products. Additionally, while the ratio of added sugar to NNS quantities supplied by beverages decreased 90 % in HIC and 11 % in UMIC, this ratio increased by 33 % in LMIC. These divergences between amounts of added sugars and NNS sold in different country income categories is consistent with previous work^(4,5)^. Baker *et al.* demonstrated that the amount of added sugar sold in beverages declined in HIC but increased in LMIC and UMIC, while the amount of NNS sold in beverages increased in all country income categories^(4)^. The growth of NNS and added sugars in LMIC and UMIC could reflect the overall increased supply of UPF in the Global South, associated with the ‘nutrition transition’^(4)^. These findings may also reflect the higher number of policy actions to improve population health and a ‘double standard’ of transnational corporation’s product reformulation activities, with more commitments to provide reformulated, ‘better for you’ products in HIC than in middle-income countries^(36)^.

Although there are at least twelve sweeteners used by food and beverage manufacturers globally, sorbitol had the highest g/capita sales in the global food supply. However, as this sweetener is only 0·6x sweeter than sucrose, a larger amount is used to achieve a sweet taste when compared with sweeter NNS (see Table 2). Stevia had the highest rates of growth in all regions with available data for this sweetener. However, as stevia is still a novel sweetener relative to other NNS, approvals for its use in the food supply for several regions occurred after the start of our data set. Illustratively, stevia was approved for use in Australia and New Zealand in 2008 and in Europe in 2011^(37)^. This may, in part, contribute to the rapid growth of stevia sales observed in our results. However, this result is in line with other literature reporting increases in the use of stevia in areas including Latin America and Australia^(31,32)^.

Our results suggest that the sweetness of the global beverage supply has decreased over time. Consistent with previous research, the greatest contributor to the decreasing sweetness of beverages was a decrease in the amount of added sugars sold in HIC, where beverage sales are highest^(4,5)^. Comparatively, the quantity of added sugars sold in beverages increased in LMIC and UMIC. Additionally, the quantity of NNS sold in beverages increased in all income categories and almost all regions, except South Asia, the Middle East and North Africa and sub-Saharan Africa. The overall sweetness of packaged foods has increased over time. This trend mainly reflects increased added sugar sales in UMIC and LMIC, with limited change in HIC. Compared to policy actions that target beverages, far fewer addressed foods high in added sugars and many more were voluntary. Though a reduction in added sugar consumption is an important component of improving diets, the use of NNS to achieve this outcome may have adverse unintended consequences. First, there remains ongoing uncertainties about associations between NNS consumption and health outcomes. Second, there is a possible indirect, adverse, unintended consequence resulting from shifts in population taste preferences to sweeter palates. Third, a health halo may be ascribed to UPF containing NNS, which may then be substituted for nutritious whole foods^(38)^.

### What is driving changes in sweetener use?

Consistent with previous research, the regions with the most policy actions to reduce added sugar intakes were Europe and Central Asia, Latin America and the Caribbean, and East Asia and the Pacific^(39,40)^. These regions all had increases in NNS sales supplied by beverages, with increases in NNS sales supplied by foods in Europe and Central Asia and East Asia and the Pacific. However, an increased number of total policy actions, and most policy actions generally, did not equate to a larger magnitude of change in added sugars in food or beverages, or NNS sales supplied by food. A significant correlation was observed between increased NNS sales supplied by beverages and advertising regulations. This is consistent with research from Ricardo *et al.,* who found increased NNS use in food and beverages following the implementation of the Chilean Advertising restrictions regarding sugar^(41)^. This policy action is part of a suite of regulatory activities, including front-of-pack labeling and restrictions for institutional food/beverages sales and provisions.

For the purposes of this analysis, we measured only the number of policy actions implemented in each region. The interactions of the food system broadly, and the specific parameters of each policy action, are important contextual factors that need to be considered when interpreting this data. There are several other factors that may influence the use of NNS as a sweetening agent in packaged food and beverages. Manufacturers can use NNS as a replacement for sugar to reduce penalties from negative taxes and labeling schemes^(42)^. Illustratively, in tandem with the implemented 2018 SSB tax in the United Kingdom, manufacturer Britvic Plc. claimed that 99 % of its brand portfolio would be below the sugar content threshold, or tax exempt, by 2019^(42)^. Coca Cola and PepsiCo have made similar sugar reduction pledges globally^(43,44)^. In the USA, an increase in the use of NNS occurred immediately prior to the release of updated national labeling guidelines, which included a declaration of the grams of added sugars on the nutrition information panel^(45)^. As stated by the Global Product Manager of Cargill ‘*You have the implications across a number of countries with sugar taxations, so from a government standpoint that’s having an influence on the product development decision process*’^(45)^.

Most policy actions implemented globally targeted the food environment (the spaces in which food choices are made) and behavior change (education and skills to encourage consumers to change their behaviors), rather than addressing the overarching social, commercial and environmental determinants of health^(46)^. Given the multifactorial nature of poor diets (of which added sugar consumption is a component), and the need for dynamic suite of policies with empowered multisectoral governance structures, protections against conflicts of interest, and monitoring, accountability and enforcement mechanisms, current policy responses to reduce added sugar intakes and improve diets are inadequate in both scope and strength^(5,39)^.

Other factors that may influence increased NNS use include consumer demand^(42,47)^ and technological advances in NNS manufacturing. Increasingly, consumers are concerned about health and weight gain, particularly from added sugars^(45)^. Excess added sugar consumption has been linked to health issues including obesity and non-communicable disease^(9,48)^ and is increasingly demonized as an isolated nutrient in the media^(49,50)^. Simultaneously, consumption of NNS has increased over time, particularly in the USA^(13)^. However, low-carb, low-sugar diets may not translate to increased whole-food consumption. Instead, the market for UPF that have either small amounts or no sugar has grown in response to this consumer demand^(45)^. These market changes promote ‘permissible indulgence’ – seemingly indulgent foods with low energy content^(42)^. This has been particularly true during the coronavirus pandemic, during which consumers have increased purchasing of comfort foods^(42)^.

The development of new types of sweeteners, methods for their development and sweetener blends increases the ability of manufacturers to use them^(42)^. In several countries, NNS innovations have been increasingly approved by national and international food-regulating bodies^(37)^. Technological barriers to NNS use include mimicking the taste of sucrose, non-sucrose flavor profiles (e.g. bitterness), mouth-feel, solubility and stability^(50)^. To overcome these issues, sweetener blends are increasingly developed and used by manufacturers^(45)^. In packaged food products, added sugars provide functions other than sweetness, including structure and texture. Thus, outside of beverages, NNS are often used in tandem with added sugars, not as a replacement^(32)^. The increased quantities of NNS sold in beverages, rather than from packaged food, is reflected in the results of this study.

### Strengths and limitations

This is the first study to describe longitudinal global trends in the amount of added sugar and NNS sold in packaged foods and beverages, and to compare these trends to regional policy actions to reduce added sugar consumption. By differentiating between regions, product categories and sweetener varieties, we have been able to demonstrate differences that have not been addressed in previous research. Our study can be used to demonstrate potential secondary consequences of reductionist policy actions and may be used to help inform regulatory approaches to attenuate excess added sugar consumption and poor population diets and health outcomes.

Limitations of the study included the exclusion of low-income countries from the analysis due to a lack of sales volume data. Additionally, as we have used the sales of added sugars and NNS as a proxy for consumption in our analysis, our results are an approximation. However, given the poor recording of NNS consumption in several national dietary intake surveys, and the extended time periods between these surveys, the use of sales volume data was necessary. By using Euromonitor data, we have reported only ingredients from formal sales of packaged foods and beverages. This may under-represent true quantities, as sales via informal channels were not captured. These results should therefore be interpreted with caution.

Furthermore, we were not able to differentiate between the contribution of reformulation and overall increased purchasing of products containing added sugars and NNS to the trends observed with the data available. The policy actions included in our study were sourced from the NOURISHING database, which does not capture all policy actions implemented globally. Due to the limited number of policy actions implemented globally, an analysis at the country level was not appropriate and instead we grouped policy actions across regions. We acknowledge the complexity of assessing the impact of policy actions, which may have been implemented at varying levels at different time points, on NNS and added sugar sales and that our analysis has not accounted for other potential confounding factors. Additionally, our results do not demonstrate a comparison of NNS and added sugar sales before and after implementation of policy actions; rather, we present a rudimentary association between policy action presence and changes in the food supply. The implications of specific combinations of policy actions, lag times between implementation and impact, and the magnitude of their influence on the food supply were not captured in our study, though they are important factors that should be considered when interpreting our findings.

## Conclusion

The per capita quantity of NNS sold in beverages is increasing globally, particularly for carbonates and bottled water, though it has remained stable for food. This increase is predominantly related to increasing sales of the natural sweetener stevia and is significantly correlated with the presence of advertising restrictions for unhealthy food, food standards in public institutions and policy actions to increase the availability of health food. Comparatively, both the ratio of added sugar and NNS use, and the per capita amounts of added sugars sold in beverages, are decreasing in HIC, though per capita sales are increasing in both UMIC and LMIC, suggesting a disparity of reformulation efforts between country income categories. Added sugar sales supplied by food are increasing globally. Overall, the global packaged food supply is getting sweeter. The increased use of NNS should be monitored in national dietary intake surveys and evaluated to determine their contribution to the global burden of disease. The health and dietary impacts of this increasingly sweet food supply, and increased NNS intakes, may present a significant concern for public health in the future.

## Supplementary material

For supplementary material accompanying this paper visit https://doi.org/10.1017/S1368980022001598. click here to view supplementary material
