# Supplementary material for: Global trends in added sugars and non-nutritive sweetener use in the packaged food supply: drivers and implications for public health
Source: Public Health Nutr. 2022 Jul 28;26(5):952–64. doi: 10.1017/S1368980022001598 (PMC10346066; doi:10.1017/S1368980022001598)
Supplement: Supplementary file 1 [file S1368980022001598sup001.zip › S1368980022001598sup003.pdf]

Policy action	NNS sales supplied by beverages		Added sugar sales supplied by beverages		NNS sales supplied by packaged food		Added sugar sales supplied by packaged food	
	Spearman Correlation Coefficient	P value	Spearman Correlation Coefficient	P value	Spearman Correlation Coefficient	P value	Spearman Correlation Coefficient	P value
Advertising regulations	0.777	0.020*	-0.618	0.070	-0.299	0.258	-0.458	0.151
Food standards in public institutions	0.727	0.032*	-0.564	0.094	0.127	0.393	-0.109	0.408
Increase availability of healthier food	0.791	0.017*	-0.791	0.017*	-0.158	0.367	-0.474	0.141
Labelling regulations	0.076	0.436	0.057	0.452	0.454	0.153	0.435	0.165
Nutrition standards for public procurement	0.474	0.141	-0.316	0.245	0.000	0.500	-0.158	0.367
Public awareness campaigns	-0.092	0.423	-0.501	0.126	0.154	0.371	-0.116	0.402
Taxes	0.146	0.378	0.055	0.454	0.382	0.199	0.109	0.408
Voluntary reformulation	0.356	0.216	-0.089	0.425	0.757	0.024*	0.401	0.186
All policy actions	0.679	0.047*	-0.429	0.169	0.250	0.294	0.071	0.440
