# Supplementary material for: Global trends in added sugars and non-nutritive sweetener use in the packaged food supply: drivers and implications for public health
Source: Public Health Nutr. 2022 Jul 28;26(5):952–64. doi: 10.1017/S1368980022001598 (PMC10346066; doi:10.1017/S1368980022001598)
Supplement: Supplementary file 1 [file S1368980022001598sup001.zip › S1368980022001598sup004.pdf]

Region	Non-nutritive sweetener	Sales volume (g/capita)		% Change
		2007	*2009*	
World	Acesulfame K	0.9	1.1	↑ 23%
	Aspartame	2.9	2.7	↓ 5%
	Cyclamate	0.5	0.4	↓ 18%
	Erythritol	1.0	5.5	↑ 451%
	Maltitol syrup	14.1	15.5	↑ 10%
	Mannitol	3.0	2.6	↓ 13%
	Saccharin	0.6	0.6	↓ 5%
	Sorbitol	39.9	38.1	↓ 4%
	Stevia	0.0	0.2	n/a
	Sucralose	0.3	0.6	↑ 81%
	Xylitol	6.1	6.2	↑ 1%
East Asia and the Pacific	Acesulfame K	0.6	0.8	↑ 29%
	Aspartame	0.8	1.0	↑ 31%
	Cyclamate	0.0	0.0	↑ 8%
	Erythritol	1.1	4.1	↑ 264%
	Maltitol	4.1	4.6	↑ 13%
	Maltitol syrup	2.0	2.3	↑ 18%
	Mannitol	1.2	1.3	↑ 2%
	Saccharin	0.1	0.1	↑ 27%
	Sorbitol	25.7	31.6	↑ 23%
	Stevia	0.0	0.1	↑ 1159%
	Sucralose	0.3	0.4	↑ 53%
	Xylitol	9.4	10.7	↑ 14%
Europe and Central Asia	Acesulfame K	2.4	3.1	↑ 30%
	Aspartame	7.3	8.5	↑ 18%
	Cyclamate	2.1	1.8	↓ 12%
	Erythritol	1.7	2.2	↑ 27%
	Maltitol	29.1	34.2	↑ 18%
	Maltitol syrup	8.6	11.2	↑ 29%
	Mannitol	6.8	6.7	↓ 2%
	Saccharin	3.0	2.9	↓ 4%
	Sorbitol	84.7	87.3	↑ 3%
	Stevia	0.0	0.3	n/a
	Sucralose	0.6	1.2	↑ 92%
	Xylitol	12.0	11.6	↓ 3%
Latin America and the Caribbean	Acesulfame K	3.0	4.2	↑ 41%
	Aspartame	8.0	7.7	↓ 4%
	Cyclamate	3.2	2.6	↓ 18%
	Erythritol	0.2	1.6	↑ 553%
	Maltitol	7.9	11.6	↑ 46%
	Maltitol syrup	1.3	1.5	↑ 18%
	Mannitol	5.2	4.8	↓ 8%

	Saccharin	1.3	1.2	↓ 1%
	Sorbitol	62.8	53.7	↓ 14%
	Stevia	0.0	0.3	n/a
	Sucralose	0.9	2.7	↑ 189%
	Xylitol	4.7	4.9	↑ 4%
Middle East and North Africa	Acesulfame K	0.4	0.4	↓ 1%
	Aspartame	1.2	1.2	n/a
	Cyclamate	0.1	0.1	↓ 2%
	Erythritol	0.2	0.4	↑ 58%
	Maltitol	4.2	6.3	↑ 50%
	Maltitol syrup	0.5	0.7	↑ 51%
	Mannitol	1.0	1.2	↑ 18%
	Saccharin	0.3	0.3	↓ 3%
	Sorbitol	13.9	16.3	↑ 18%
	Stevia	0.0	0.0	n/a
	Sucralose	0.2	0.2	↑ 18%
	Xylitol	0.6	0.6	↑ 8%
North America	Acesulfame K	3.5	3.1	↓ 12%
	Aspartame	19.9	11.7	↓ 41%
	Cyclamate	0.2	0.1	↓ 33%
	Erythritol	6.2	77.6	↑ 1145%
	Maltitol	90.3	80.2	↓ 11%
	Maltitol syrup	18.6	33.3	↑ 79%
	Mannitol	21.0	15.5	↓ 26%
	Saccharin	1.7	1.2	↓ 27%
	Sorbitol	228.5	177.3	↓ 22%
	Stevia	0.0	2.2	n/a
	Sucralose	1.4	2.4	↑ 74%
	Xylitol	15.1	13.4	↓ 12%
South Asia	Acesulfame K	0.0	0.1	↑ 129%
	Aspartame	0.1	0.3	↑ 142%
	Cyclamate	0.0	0.0	n/a
	Erythritol	0.1	0.1	n/a
	Maltitol	0.3	0.5	↑ 44%
	Maltitol syrup	0.3	0.5	↑ 67%
	Mannitol	0.1	0.2	↑ 54%
	Saccharin	0.0	0.1	↑ 185%
	Sorbitol	2.3	4.7	↑ 104%
	Sucralose	0.0	0.0	n/a
	Xylitol	0.2	0.4	↑ 145%
Sub-Saharan Africa	Acesulfame K	0.0	0.0	n/a
	Aspartame	0.0	0.0	n/a
	Cyclamate	0.0	0.0	n/a
	Erythritol	0.0	0.0	n/a

	Maltitol	0.0	0.0	n/a
	Maltitol syrup	0.0	0.0	n/a
	Mannitol	0.0	0.0	n/a
	Saccharin	0.0	0.0	n/a
	Sorbitol	0.0	0.0	n/a
	Sucralose	0.0	0.0	n/a
	Xylitol	0.0	0.0	n/a
HIC	Acesulfame K	3.2	3.7	↑ 15%
	Aspartame	12.2	10.6	↓ 13%
	Cyclamate	1.5	1.3	↓ 13%
	Erythritol	3.8	27.8	↑ 639%
	Maltitol	52.4	51.2	↓ 2%
	Maltitol syrup	15.1	21.5	↑ 43%
	Mannitol	12.4	9.9	↓ 21%
	Saccharin	2.6	2.4	↓ 10%
	Sorbitol	143.1	119.2	↓ 17%
	Stevia	0.0	1.0	↑ 33461%
	Sucralose	1.0	1.8	↑ 75%
	Xylitol	21.4	17.0	↓ 21%
UMIC	Acesulfame K	0.9	1.2	↑ 35%
	Aspartame	1.8	1.9	↑ 9%
	Cyclamate	0.5	0.4	↓ 16%
	Erythritol	0.9	3.7	↑ 322%
	Maltitol	5.7	8.6	↑ 51%
	Maltitol syrup	0.9	1.4	↑ 56%
	Mannitol	2.3	2.7	↑ 14%
	Saccharin	0.4	0.4	↑ 4%
	Sorbitol	31.5	36.2	↑ 15%
	Stevia	0.0	0.1	↑ 2907%
	Sucralose	0.3	0.7	↑ 112%
	Xylitol	5.6	8.1	↑ 44%
LMIC	Acesulfame K	0.2	0.2	↑ 36%
	Aspartame	0.4	0.7	↑ 50%
	Cyclamate	0.1	0.1	↓ 15%
	Erythritol	0.2	0.3	↑ 67%
	Maltitol	1.1	1.4	↑ 25%
	Maltitol syrup	0.6	0.9	↑ 36%
	Mannitol	0.3	0.4	↑ 24%
	Saccharin	0.1	0.1	↑ 24%
	Sorbitol	12.7	17.8	↑ 40%
	Stevia	0.0	0.0	n/a
	Sucralose	0.0	0.1	↑ 112%
	Xylitol	1.3	1.9	↑ 47%
