# Supplementary material for: Global trends in added sugars and non-nutritive sweetener use in the packaged food supply: drivers and implications for public health
Source: Public Health Nutr. 2022 Jul 28;26(5):952–64. doi: 10.1017/S1368980022001598 (PMC10346066; doi:10.1017/S1368980022001598)
Supplement: Supplementary file 1 [file S1368980022001598sup001.zip › S1368980022001598sup005.pdf]

**Region**	**Lower-Middle income countries ($1,026 - $3,995)***	**Upper-Middle income countries ($3,996 - $12,375)***	**High income countries (>$12,376)***
East Asia and Pacific	Indonesia	China	Australia
	Philippines	Malaysia	Hong Kong
	Viet Nam	Thailand	Japan
			Singapore
			South Korea
			Taiwan
			New Zealand
Europe and Central Asia	Georgia	Azerbaijan	Austria
	Ukraine	Belarus	Belgium
	Uzbekistan	Bosnia and Herzegovina	Czech Republic
		Bulgaria	Denmark
		Croatia	Estonia
		Kazakhstan	Finland
		North Macedonia	France
		Romania	Germany
		Russian Federation	Greece
		Serbia	Hungary
		Turkey	Ireland
			Italy
			Latvia
			Lithuania
			Netherlands
			Norway
			Poland
			Portugal
			Slovenia
			Slovakia
			Spain
			Sweden
			Switzerland
			United Kingdom
Latin America and Caribbean	Bolivia	Argentina	Chile
	Guatemala	Brazil	Uruguay
		Colombia	
		Costa Rica	
		Dominican Republic	
		Ecuador	
		Mexico	

		Peru	
Middle East and North Africa	Egypt<br>Morocco<br>Tunisia	Algeria	Israel<br>Qatar<br>Saudi Arabia<br>United Arab Emirates
North America			Canada<br>USA
South Asia	India<br>Pakistan		
Sub-Saharan Africa	Cameroon<br>Kenya<br>Nigeria	South Africa	
