# Supplementary figures and images for: Global trends in added sugars and non-nutritive sweetener use in the packaged food supply: drivers and implications for public health
Source: Public Health Nutr. 2022 Jul 28;26(5):952–64. doi: 10.1017/S1368980022001598 (PMC10346066; doi:10.1017/S1368980022001598)

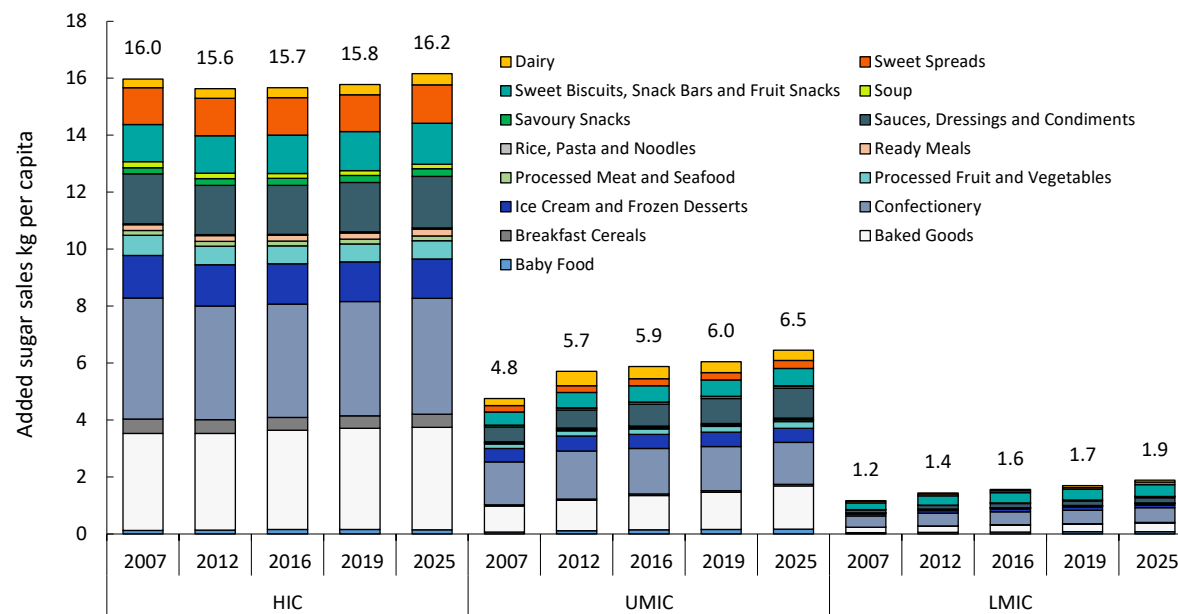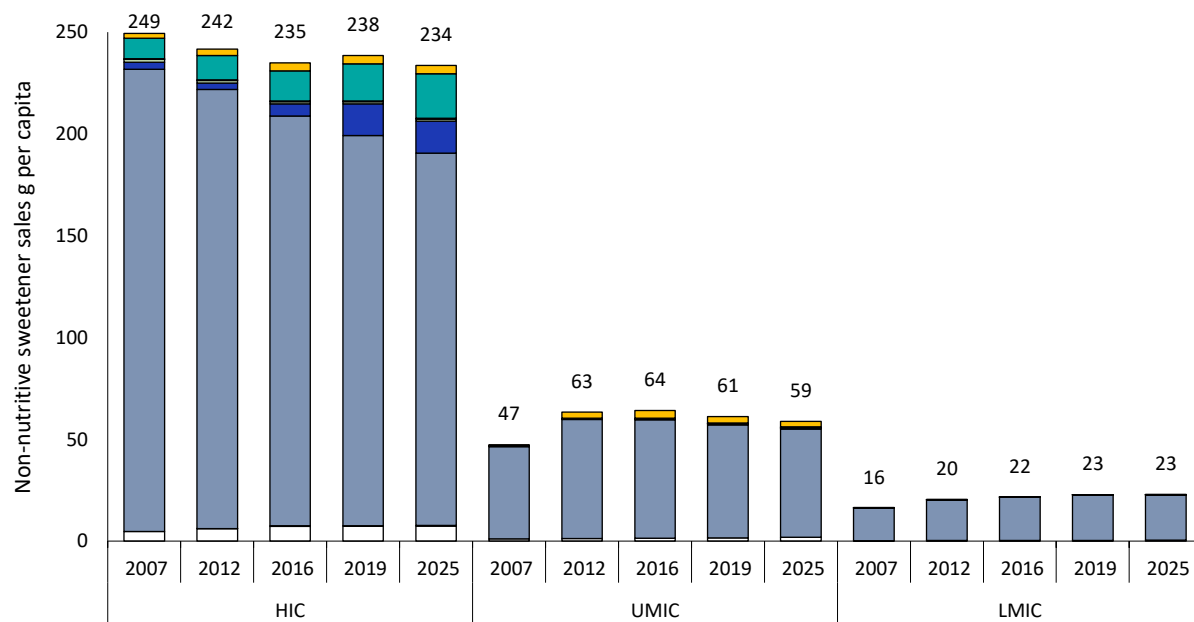

Supplement: Supplementary file 1 [file S1368980022001598sup001.zip › S1368980022001598sup001.pdf]

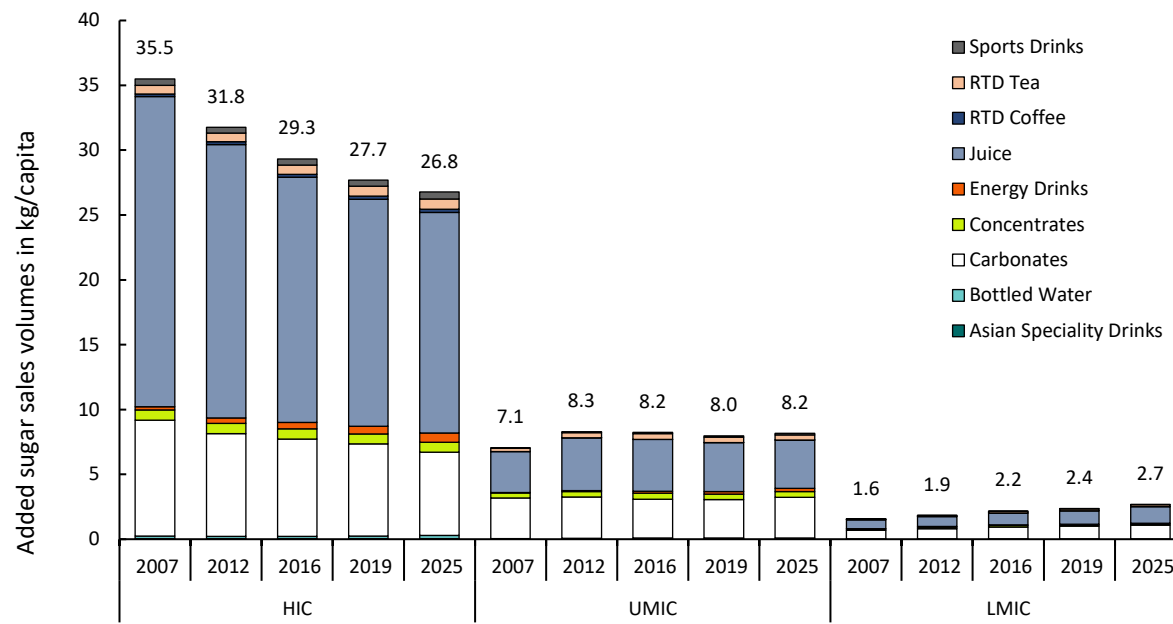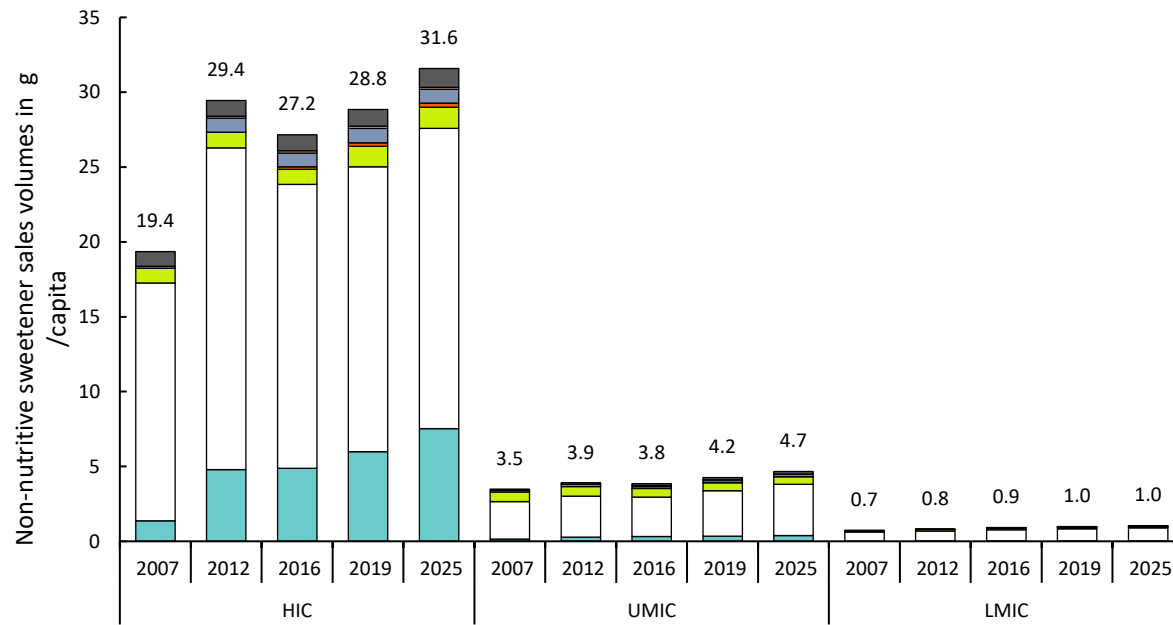

Supplement: Supplementary file 1 [file S1368980022001598sup001.zip › S1368980022001598sup002.pdf]
